# Supplementary material for: Diagnostic and prognostic roles of circulating miRNA-223-3p in hepatitis B virus–related hepatocellular carcinoma
Source: PLoS One. 2020 Apr 24;15(4):e0232211. doi: 10.1371/journal.pone.0232211 (PMC7182200; doi:10.1371/journal.pone.0232211)
Supplement: S1 Table — (DOCX) [file pone.0232211.s001.docx]

**Supplement Table1.** Clinicopathological features of 4 patients undergoing surgical resection

| **Baseline Characteristics** | **Case 1** | **Case 2** | **Case 3** | **Case 4** |
| --- | --- | --- | --- | --- |
| Age (years) | 59 | 51 | 48 | 52 |
| Gender | Male | Female | Male | Male |
| Aspartate aminotransferase (IU/L) | 47 | 24 | 27 | 48 |
| Alanine aminotransferase (IU/L) | 52 | 15 | 30 | 97 |
| Serum albumin (g/dL) | 3.9 | 3.8 | 3.9 | 4.4 |
| Total bilirubin (mg/dL) | 0.5 | 0.5 | 0.9 | 0.4 |
| Platelet count (10^9^/L) | 381 | 232 | 245 | 130 |
| HBeAg positivity | Positive | Negative | Negative | Negative |
| Log_10_ HBV DNA (IU/mL) | 5.1 | undetectable | 3.7 | 3.1 |
| Alpha fetoprotein (ng/mL) | 7173.8 | 1.70 | 350.5 | 175.0 |
| Presence of cirrhosis | Yes | No | Yes | Yes |
| Tumor size (cm) | 2.9 | 2.6 | 1.9 | 3.0 |
| Tumor differentiation | Poor | Moderate | Moderate | Well |
| Microvascular invasion | Yes | No | Yes | No |
| BCLC stage | A | A | 0 | A |
